# Predictors of primary and secondary sexual abstinence among never-married youth in urban poor Accra, Ghana

**DOI:** 10.1186/s12978-020-0885-4

**Published:** 2020-02-21

**Authors:** Nurudeen Alhassan, F. Nii-Amoo Dodoo

**Affiliations:** 1 African Institute for Development Policy (AFIDEP), Area 6, Plot 6/3, P. O. Box, 31024 Lilongwe, Malawi; 2 The Pennsylvania State University, Pennsylvania, USA; 3 University of Ghana, Accra, Ghana

**Keywords:** Primary sexual abstinence, Secondary abstinence, Urban poor, Youth, Accra, Ghana

## Abstract

**Background:**

Sexual abstinence is a key component of the strategy to address unwanted pregnancies, sexually transmitted infections and HIV among youth in sub-Sahara Africa. But contextual pressures make abstaining from sex a formidable task for urban poor youth in the sub-region. Nevertheless, some youth in these settings still manage to resist the pressure to initiate sex early, while others choose abstinence after an initial sexual debut. Few studies in the sub-region have examined sexual abstinence among urban poor youth. We therefore examined the factors that predict primary and secondary sexual abstinence among youth in urban poor Accra. The findings highlight the protective factors associated with sexual intercourse and should help to address the needs of youth at risk of unprotected sex.

**Methods:**

The study analysed pooled data from two rounds of the Urban Health and Poverty Survey. The surveys analysed were conducted in 2011 and 2013. The analysis was restricted to unmarried youth between age 20 and 24 years. The total sample comprised 235 female and male youth. We conducted multinomial logistic regression analysis to examine the predictors of primary and secondary abstinence relative to current sexual intercourse.

**Results:**

The results showed that being female, sexual communication with only fathers, sexual communication with only friends and slum residence were negatively associated with primary sexual abstinence. Contrarily, being in school, attaching importance to religion, residing in a household that received social support and sexual communication with both parents were positively associated with primary abstinence. Regarding secondary abstinence, only the sexual communication variables had significant effects. Sexual communication with both parents positively predicted secondary abstinence while communication with fathers-only and friends-only had a negative effect.

**Conclusion:**

Sexual abstinence is predicted by factors which range from individual through household factors to the locality of residence. Despite the importance of all the predictors, the study found that sexual communication with both parents was the only factor that predicted a higher likelihood of both primary and secondary sexual abstinence. We therefore recommend sexual communication between parents and youth as a key strategy for promoting sexual abstinence among urban poor youth in Accra, Ghana.

## Plain English summary

Abstaining from sex is one of the most effective ways to prevent unwanted pregnancies and sexually transmitted infections among youth in sub-Sahara Africa. However, youth in urban poor areas of the sub-region find it more difficult to abstain from sex than their peers in rural and non-poor urban areas. Despite this difficulty, some youth in urban poor areas manage to abstain from sex. Unfortunately, studies designed to understand the factors that influence sexual abstinence among these youth are sparse. Our objective in this study was to identify the factors that influence sexual abstinence among urban poor youth in Accra, Ghana. We analysed data from a survey conducted by the Regional Institute for Population Studies at the University of Ghana. We found that females, youth who talk about sex with only their fathers, those who talk about sex with only their friends and those living in a slum were less likely to abstain from sex. However, youth in school, those who attached importance to religion, those in households that received social support and youth who talked about sex with both their mothers and fathers were more likely to abstain from sex. In conclusion, sexual abstinence among urban poor youth in Accra is influenced by gender, schooling, religion, household support, communication about sex with both parents, fathers-only and only friends as well as residence in a slum. These findings suggest that efforts to promote sexual abstinence among youth in urban poor Accra need to prioritise these factors to be effective. Sexual communication with both parents especially needs to be prioritised in interventions as it was the only factor that predicted a higher likelihood of never engaging in sexual intercourse or practising abstinence after sexual debut.

## Background

The onset of sexual intercourse is associated with adverse sexual and reproductive health outcomes including unintended pregnancies, sexually transmitted infections (STIs) and HIV/AIDS [1, 2]. In Ghana, the proportion of unmarried youth between ages 15 and 24 years that have initiated sexual intercourse increased significantly over the last decade [3, 4]. About 78% of unmarried females and 74% of unmarried males are sexually active by 20–24 years [4]. In spite of the increasing proportion of youth engaging in sexual intercourse, contraceptive use remains quite low. For instance, only 35% of sexually active unmarried females between ages 20 and 24 years use modern contraception [4]. It is therefore no coincidence that unintended pregnancies, STIs and HIV/AIDS are highest among youth compared to other population sub-groups in the country. It is estimated that 26% of new HIV cases occur among youth aged 15–24 years [5].

Research in sub-Saharan Africa shows that adolescents and youth in urban poor settings have an increased risk of initiating sexual intercourse early, having multiple sexual partners, experiencing unintended pregnancies and contracting sexually transmitted infections [6–9]. For instance, a study by Kabiru et al., in Nairobi found that the median age at sexual debut was 18 years for adolescents in non-slum areas and 15 years for those living in slums [9]. In another study in Nairobi, Beguy et al., found that 61% of sexually active girls in slums aged 14–22 years had ever been pregnant [6]. In Ghana, it is reported that as much as 80% of girls in Ga Mashie, an urban poor setting in Accra, become pregnant before age 22 years [10]. Urban poverty provokes adolescents and youth especially females into sexual relationships to support themselves and their families [11, 12]. Beyond low incomes, this is also a result of high unemployment, unstable wages, crime, poor educational facilities, and the lack of recreational facilities in these settings [6]. In sum, urban poor youth in the sub-region are less likely to abstain from sexual intercourse than youth in the general population, even though abstinence is one of the most effective strategies for preventing both unwanted pregnancies and STIs including HIV.

Notwithstanding the vulnerability of urban poor youth to sexual intercourse, there are youth in these settings who abstain from sex despite the social and economic pressures to have sex. Yet, few studies have investigated these youth to understand the factors that protect them from engaging in sexual intercourse in such risky settings [13, 14]. Among studies that have examined sexual abstinence, only few have simultaneously studied the factors associated with primary and secondary abstinence [15]. Many have focused exclusively on primary sexual abstinence even though some youth commit to abstinence after sexual debut due to negative experiences at first sex or because of regrets about that debut encounter.

This study, therefore, seeks to contribute to the scant literature on youth sexual abstinence in urban poor sub-Saharan Africa by examining the predictors of primary and secondary abstinence among urban poor youth in Accra, Ghana. Secondly, understanding the factors that predict sexual abstinence in urban poor settings may provide reproductive health practitioners and policy makers with valuable insights that can contribute to developing effective interventions to delay sexual debut and reduce teenage pregnancies and STIs. This study should however not be misconstrued as encouraging abstinence as the only effective option for preventing unwanted pregnancies and STIs among urban poor youth in the sub-region. Other components of the ABC (Abstain, Be faithful and use Condoms) approach especially condom use are equally effective for preventing unwanted pregnancies, STI and HIV/AIDS. It is also important to point out that the study defined abstinence exclusively as refraining from penetrative vaginal sex.

**Fig. 1:**
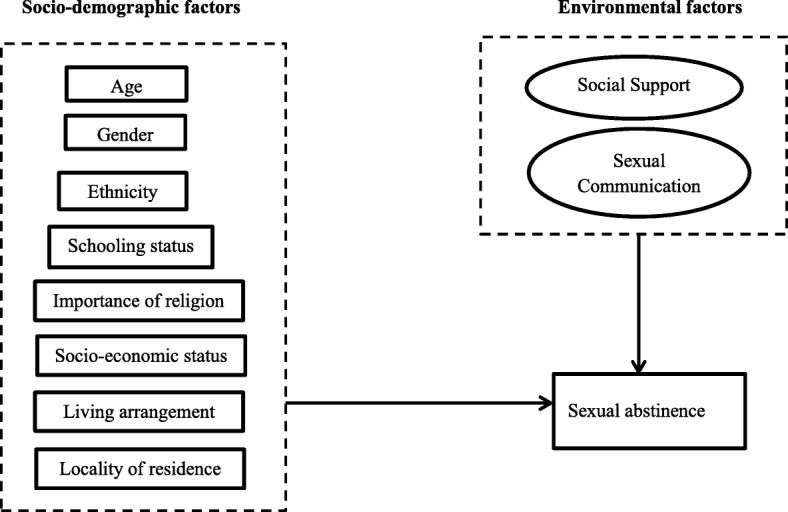
Conceptual framework of the predictors of youth sexual abstinence in urban poor Accra

The conceptual framework which informed the selection of predictor variables for this study was based on the integrative theoretical framework of adolescent sexual abstinence [16]. This theoretical framework argues that sexual abstinence among adolescents and youth is predicted by socio-demographic, environmental and psychological factors [16]. The socio-demographic predictors of sexual abstinence in the integrative theoretical framework include age, gender, ethnicity and the importance of religion [16]. The environmental factors in the framework are parental support, rules and monitoring while the psychological factors include beliefs and norms regarding abstinence. The psychological factors in the theoretical framework were not available in the secondary data used for this study. We therefore adapted the framework to focus on the socio-demographic and environmental predictors of sexual abstinence. Beyond the variables in the framework, we also included other socio-demographic and environmental factors that have been shown in the literature to predict adolescent and youth sexual abstinence behaviour (Fig. 1).

The relationship between the socio-demographic factors in the conceptual framework and sexual abstinence is well-documented in the literature. With regards to age, for instance, studies show that a disproportionately higher number of sexually abstinent youth are younger than their sexually active counterparts [1, 9, 17–19]. Age is generally associated with physical maturation and hormonal changes which increase the urge to engage in sexual intercourse [1]. On gender, studies conducted in the United States and Western Europe show that male adolescents and youth are less likely to abstain from sexual intercourse than females [20, 21]. In Ghana, however, female adolescents are less likely to abstain from sex compared to their male counterparts [18, 22]. The pattern in Ghana can be attributed to gender inequities and social norms that compel females to initiate sexual intercourse early, usually with older males, for financial and other related support [23]. Regarding family composition and living arrangement, dual-parent families tend to be more amenable to adolescent sexual abstinence than single-parent families [20, 24]. This is partly because dual-parent families have better supervision and economic advantage over single-parent families.

With regards to the environmental factors, adolescent and youth sexual communication with parents has received considerable attention in the abstinence literature. But the evidence on the relationship between sexual communication and abstinence is inconclusive. While some studies find parental sexual communication to be associated with a higher likelihood of abstinence [25, 26], others find the reverse [27, 28]. Many of the studies on sexual communication also focus on mothers [28–31], while paying less attention to the effect of communicating with fathers and friends. Although social support is less-commonly examined, few studies have begun to examine the effect of cash transfers on adolescent and youth sexual behaviour [32, 33]. One study in South Africa found that young women who received cash transfer were less likely to engage in unprotected sex [33]. Another study in South Africa, however, found that the effect of cash transfers on sexual behaviour was short-term as adolescents reverted to previous sexual behaviour with the end of cash transfer programmes [32].

In conclusion, even though there is overwhelming evidence on the predictors of adolescent and youth sexual abstinence behavior, few of the studies focus on young people in urban poor sub-Saharan Africa. Consequently, the effect of the factors identified in the conceptual framework on sexual abstinence among urban poor youth in the sub-region is less clear. Research is therefore needed to assess how factors such as gender, sexual communication and informal social support influence sexual abstinence in urban poor sub-Saharan Africa, where abstinence from sex is a formidable task.

## Data and methods

### Study context

This study was conducted in three urban poor communities in central Accra—James Town, Ussher Town and Agbogbloshie. The inhabitants of James Town and Ussher Town are predominantly of the Ga ethnic origin, the indigenous people of Accra. These two communities are located along the coast of Accra on the Gulf of Guinea, with fishing and fish mongering serving as important economic activities. Agbogbloshie, on the other hand, is a slum migrant community adjoining the traditional enclaves of James Town and Ussher Town. Agbogbloshie is home to one of the biggest markets in central Accra, and most of its inhabitants are engaged in petty trading.

The three communities are densely populated due to high birth rates, and also because they provide cheap housing options to migrants to the city. The communities are characterised by poor socio-economic conditions such as poor sanitation, health and education. There is evidence that the poor socio-economic conditions in the communities expose inhabitants to poor sexual and reproductive health influences. One anthropological study conducted in James Town and Ussher Town documented that women used pregnancy and childbearing as a strategy to overcome economic hardships by depending on men who impregnated them for financial support [34]. Sexual relations between young girls and economically viable older men are also thought to be subtly encouraged as a means of survival. This situation has resulted in widespread teenage pregnancy and early child bearing in the communities.

These three communities were selected by the Regional Institute for Population Studies (RIPS) at the University of Ghana for the Urban Health and Poverty Project because of their poor socio-economic conditions and poor health outcomes. The current study focused on the three communities because the data relied on was limited to those communities. Secondly, adolescents and youth in the three communities are known to initiate sexual intercourse much earlier, and teenage pregnancy is commonplace [22, 35]. The context of the three communities therefore provides an opportunity to study sexual abstinence in an environment where abstinence practice is extremely difficult.

### Data

The study relied on secondary data from the Urban Health and Poverty Surveys. The Urban Health and Poverty Surveys were conducted by RIPS, University of Ghana. The surveys were undertaken in the three communities mentioned above, and sought to generate data on the relationships between population, poverty and health in the urban setting. In all, three surveys were conducted. The first survey was conducted in 2010, the second in 2011 and the third in 2013.

A two-stage systematic sampling technique was used to select representative samples of households and individuals for the surveys. In the second and third surveys which had larger sample sizes, 29 enumeration areas (EAs) were initially sampled systematically from the three localities. The number of EAs in a locality was proportional to the size of the locality. Sixteen EAs were selected from Ussher Town, the biggest locality, with eight from James Town and five from Agbogbloshie. Forty households were then systematically sampled from each of the EAs. This means that a total of 1160 households were selected for the surveys, 640 from Ussher Town, 320 from James Town and 200 households from Agbogbloshie. Out of this number, a total of 806 and 782 household interviews were completed in the second and third surveys respectively. Of the 806 households interviewed in the second survey, 445 households were from Ussher Town, 235 from James Town and 126 households from Agbogbloshie. Similarly, 457 households from Ussher Town, 218 from James Town and 107 households from Agbogbloshie were interviewed in the third survey. All eligible (women aged 15–49 years and men aged 15–59 years) and willing individuals from the selected households were interviewed. Therefore, the number of individuals interviewed per household depended on the number eligible and willing to participate in the surveys. A total of 1010 and 790 individual interviews were completed in the second and third surveys respectively.

In this paper, we analysed data from a pooled sample of never-married youth from the second and third surveys. Data from the first survey were excluded because a number of variables were not available in that dataset. We restricted our analysis to never-married youth aged between 20 and 24 years. This is because youth in this age category have had more time to either engage in or abstain from sex compared to those aged 15–19 years. There were 122 and 113 youths from the second and third survey respectively who met our inclusion criteria. Given the small number of cases in each of these surveys, it became necessary to pool together data from both surveys to obtain a reasonable number of cases for viable statistical analyses. This resulted in a total sample of 235 never-married youth.

### Ethical procedures for the study

Before administering the survey instruments, respondents were introduced to the purpose and objectives of the surveys. Respondents were informed that participation was voluntary, and the risks and rights of participants were also highlighted. Those who volunteered to participant in the surveys were required to sign written informed consent forms. As reciprocity for participation, respondents were given household items such as cooking oil, soap, tooth paste and napkins after the interviews. These household items amounted to about US$ 5 per respondent. To guarantee anonymity, respondents’ identifiers, including names and addresses were removed before data were made available for analysis. Ethical approval for the surveys was granted by the Noguchi Memorial Institute for Medical Research—Institutional Review Board (NMIMR-IRB) at the University of Ghana.

### Measurements

#### Outcome variable

The outcome variable of this study, sexual abstinence, was derived from two questions in the survey instruments. The first question asked respondents whether they had ever engaged in sexual intercourse, defined as penile-vaginal intercourse. The second question asked respondents that had ever engaged in sexual intercourse to indicate the date of their last sexual intercourse. We categorised the youth that had never engaged in sexual intercourse as primary abstainers and those whose last sexual intercourse occurred more than a year before the survey date as secondary abstainers. The youth who had sex in the last 12 months of the survey were classified as currently sexually active. Thus, the outcome variable had three categories; primary abstinence, secondary abstinence and currently sexually active. The measurement of secondary sexual abstinence in this study is similar to the measurement used in previous studies such as the study of Kabiru and Ezeh which examined sexual abstinence among adolescents in four sub-Saharan African countries and the study of Koffi and Kawahara in Ivory Coast [15, 18].

#### Predictor variables

The factors included in the analysis as predictors of sexual abstinence were based on the conceptual framework and included age, sex, ethnicity, school attendance, importance of religion, source of sexual communication, household living arrangement, household socio-economic status, household access to social support, and locality of residence. Age was measured as a continuous variable ranging from 20 to 24 years. The sex of respondents was classified as either male or female. Regarding ethnicity, the surveys asked respondents to indicate their ethnic affiliation. Majority of the respondents were Ga/Dangmes, followed by Akans with the remaining ethnic groups forming the minority. We therefore classified ethnicity into three categories; Ga/Dangme, Akan and Other. School attendance was measured by whether the respondent was in school or out of school at the time of the surveys. In addition, the surveys asked respondents to indicate how important religion was to them. The initial response categories were; very important, fairly important, indifferent, fairly unimportant and not important at all. An analysis of the responses showed that they were heavily skewed, with three quarters of respondents indicating that religion was very important to them. We therefore recoded the initial responses into two categories; very important and fairly important.

Regarding sexual communication, the surveys asked adolescent and youth respondents to indicate (Yes or No) from a list who they would talk to about sex. The list included father, mother, uncle, aunt, grandparent(s), teachers, religious leaders etc. We computed four sources of sexual communication (fathers only, mothers only, both parents and friends only) from this list by dummy coding “Yes” responses to these four critical sources of information but “No” to all the other sources. Therefore, sexual communication with fathers means that the respondent would discuss issues about sex with their father but not with any other person in the list provided. Similarly, communication with both parents means that the respondent would only discuss issues about sex with their mother and father but not with any other person in the list.

The respondent’s household living arrangement was measured by whether they were living with their parent(s) or not. The socio-economic status of respondent’s household was measured by computing a wealth index score from household items such as refrigerators, televisions, bicycles, etc. using principal component analysis. The wealth index score was initially divided into quintiles, but later recoded into three categories; poorer, middle and richer. The respondent’s access to social support was measured by whether (Yes or No) their households had received cash or in-kind support from family members outside the household in the last 12 months before the survey. In terms of the locality of residence, we classified the respondents into the three study communities; Agbogbloshie, James Town and Ussher Town.

### Statistical analyses

Frequencies, percentages and mean were used to describe the sample and to demonstrate the differences in their characteristics by gender. Multinomial logistic regression analysis was then used to examine the factors that significantly predicted primary and secondary abstinence, after controlling for all the characteristics in Table 1 below. All the variables in the multinomial logistic regression model were entered in one step. Our reference group in the regression analysis was the currently sexually active youth. We used STATA to run all our analysis, and the results of the multinomial logistic regression analysis were presented as relative risk ratios (RRR).

**Table 1:** Characteristics of study sample and differences by gender

Characteristics	Both sexes (*n* = 235)		Males (*n* = 111)		Females (*n* = 124)	
**Outcome variable**	**Freq.**	**%**	**Freq.**	**%**	**Freq.**	**%**
Sexual status						
Primary sexual abstainers	52	22.1	30	27.0	22	17.7
Secondary sexual abstainers	100	42.6	40	36.1	60	48.4
Currently sexually active	83	35.3	41	36.9	42	33.9
**Predictor variables**						
Age (20–24 years)						
Mean (std. deviation)	21.7 years (1.41)		21.8 years (1.37)		21.6 years (1.44)	
Ethnicity	**Freq.**	**%**	**Freq.**	**%**	**Freq.**	**%**
Ga/Dangme	143	60.8	79	71.2	64	51.6
Akan	65	27.7	24	21.6	41	33.1
Other	27	11.5	8	7.2	19	15.3
School attendance						
In school	42	17.9	27	24.3	15	12.1
Out of school	193	82.1	84	75.7	109	87.9
Importance of religion						
Very important	170	72.3	78	70.3	92	74.2
Fairly important	65	27.7	33	29.7	32	25.8
Living arrangement						
Living with parent(s)	115	48.9	58	52.3	57	46.0
Not living with parent(s)	120	51.1	53	47.7	67	54.0
Household SES						
Poorer	86	36.6	36	32.4	50	40.3
Middle	44	18.7	20	18.0	24	19.4
Richer	105	44.7	55	49.6	50	40.3
Household social support	Freq.	%	Freq.	%	Freq.	%
Yes	77	32.8	36	32.4	41	33.1
No	158	67.2	75	67.6	83	66.9
Discusses sex with fathers						
Yes	16	6.8	11	9.9	5	4.0
No	219	93.2	100	90.1	119	96.0
Discusses sex with mothers						
Yes	60	25.5	25	22.5	35	28.2
No	175	74.5	86	77.5	89	71.8
Discusses sex with both parents						
Yes	58	24.7	23	20.7	35	28.2
No	177	75.3	88	79.3	89	71.8
Discusses sex with friends						
Yes	55	23.4	27	24.3	28	22.6
No	180	76.6	84	75.7	96	77.4
Locality of residence						
James Town	136	57.9	66	59.5	70	56.5
Ussher	71	30.2	32	28.8	39	31.5
Agbogbloshie	28	11.9	13	11.7	15	12.1

**Source:** Urban Health and Poverty Survey, 2011 & 2013

## Results

### Characteristics of study sample

Table 1 presents the characteristics of the study sample. The mean age of the respondents was 21.7 years. More than half (53%) of them were females, and the mean age of females was similar to that of males. The majority of the respondents (60.8%) were of the Ga/Dangme ethnic background. Few (17.9%) of them were in school, with a greater percentage of males (24.3%) in school compared to females (12.1%). Approximately three quarters of the respondents reported that religion was very important to them. Overall, less than half of the respondents were living with at least one of their parents. However, a higher percentage (52.3%) of males were living with their parents compared to females (46%). More than one-third of them were residing in poorer households, and a higher percentage of females (40.3%) were living in poorer households than males (32.4%). Similarly, about a third of the respondents were in households that had received informal social support 12 months preceding the surveys. The majority of the respondents resided in Ussher Town.

In terms of sexual intercourse, more than three-quarters of them had ever engaged in sexual intercourse. This means that less than a quarter of the respondents were primary sexual abstainers. Specifically, 22% of them had never engaged in sexual intercourse (primary abstainers) and about an additional 43% had abstained from sex for more than a year (secondary abstainers), while the remaining 35% had sex within the last 12 months. A higher percentage of females (82.3%) had ever engaged in sexual intercourse compared to males (73%). This implies that only one in five females (17.7%) were primary abstainers compared to more than a quarter of the males. Almost half of the females were secondary abstainers compared to a little over a third of males. The proportion of females (33.9%) who were currently sexually active was similar to that for males (36.9%).

Regarding sexual communication, about 7% of the respondents indicated that they would discuss issues about sex with only their father; a higher percentage of males (9.9%) reported that they would discuss issues of sex with fathers only, compared to females (4%). A quarter of the respondents indicated that they would discuss issues of sex with mothers only. A higher percentage of females (28.2%) reported that they would discuss sex related matters with their mother only, compared to males (22.5%). Similarly, a quarter of the respondents indicated that they would discuss issues about sex with both father and mother, and a higher percentage of females reported this than males. Close to a quarter of the study participants reported that they will discuss issues about sex with friends only. The percentage of males who indicated that they would discuss issues about sex with their friends was higher than females. Overall, almost 20% of the study participants reported other sources including teachers, religious leaders, grandparents, uncles and siblings as their preferred sources for discussion about sex.

### Predictors of primary and secondary sexual abstinence

Table 2 presents the results of the multinomial logistic regression analysis. The model explained 43% of the variance in sexual abstinence behaviour, suggesting a good fit with the data. Gender emerged as a significant predictor of primary sexual abstinence. Compared to being male, being female decreased the relative likelihood of being a primary sexual abstainer than currently sexually active by 66% (*p* < 0.05). Thus, females were significantly less likely to be primary sexually abstinent than currently sexually active compared to males. However, there was no significant gender difference between secondary abstainers and the currently sexually active.

**Table 2:** Predictors of primary and secondary sexual abstinence

Predictors of sexual abstinence	Primary sexual abstinence vs. Currently sexually active	Secondary abstinence vs. Currently sexually active
	Relative Risk Ratio (RRR) (Standard Error)	Relative Risk Ratio (RRR) (Standard Error)
Age	0.86 (0.16)	1.30 (0.25)
Sex		
Males (ref.)	1.00	1.00
Females	0.34* (0.17)	1.20 (0.61)
Schooling status		
Out of school (ref.)	1.00	1.00
In school	3.62* (2.17)	1.23 (0.87)
Ethnicity		
Ga/Dangme (ref.)	1.00	1.00
Akan	1.95 (1.18)	1.00 (0.64)
Other	3.24 (2.32)	0.42 (0.37)
Importance of religion		
Very important (ref.)	1.00	1.00
Fairly important	0.32* (0.18)	0.73 (0.42)
Living arrangement		
Not living with parent(s) (ref.)	1.00	1.00
Living with Parent(s)	0.76 (0.37)	0.60 (0.30)
Household wealth status		
Poor (ref.)	1.00	1.00
Middle	0.22 (0.18)	0.60 (0.41)
Rich	1.20 (0.64)	0.72 (0.43)
Household received social support		
No (ref.)	1.00	1.00
Yes	2.89* (1.40)	1.47 (0.77)
Locality of residence		
Ussher Town (ref.)	1.00	1.00
James Town	1.82 (0.93)	0.81 (0.45)
Agbogbloshie	0.12* (0.12)	0.31 (0.29)
Discusses sex with fathers		
No (ref.)	1.00	1.00
Yes	0.14* (0.13)	0.08* (0.09)
Discusses sex with both parents		
No (ref.)	1.00	1.00
Yes	12.90** (10.46)	5.82* (4.74)
Discusses sex with friends		
No (ref.)	1.00	1.00
Yes	0.11** (0.08)	0.17* (0.12)
Pseudo R^2^ 0.43		

*p < 0.05;**p < 0.01;***p < 0.001

The results show that being in school increased the relative likelihood of being a primary sexual abstainer than being currently sexually active by more than three times. Schooling, however, did not significantly differentiate the secondary abstainers from the currently sexually active youth. Furthermore, the importance of religion to youth is evidenced in it being a significant predictor of primary sexual abstinence. Compared to youth who reported that religion was very important to them, those who reported that religion was fairly important to them had a 68% reduced relative likelihood of being primary sexual abstainers compared to the currently sexually active. Nonetheless, how important religion was to youth had no statistically significant association when the secondary abstainers were compared to the currently sexually active.

Living in a household that received social support from family members positively predicted primary sexual abstinence. Compared to living in a household that did not receive social support, residing in a household that received support from family members increased the relative likelihood of being a primary sexual abstainer than the currently sexually active by more than two-fold. But, social support from family members did not distinguish secondary abstainers from currently sexually active youth. Regarding the locality of residence, living in Agbogbloshie, the slum community, decreased the likelihood primary sexual abstinence by 88% relative to living in Ussher Town. Locality had no statistically significant association when the secondary sexual abstainers were compared to the currently sexually active youth.

Communicating about issues related to sex with both parents was positively predicted with primary and secondary abstinence. Communication about sex with both parents increased the relative likelihood of being a primary abstainer compared to being currently sexually active by more than 12 times. Similarly, communication about sex with both parents increased the relative likelihood of being a secondary sexual abstainer compared to being currently sexually active by more than five times. This means that youth who discussed sex-related issues with both parents were significantly more likely to be primary abstainers or secondary abstainers than the currently sexually active.

Contrary to expectation, the results show that sexual communication with only fathers was associated with a lower likelihood of sexual abstinence. Discussing sexual issues with only fathers decreased the relative likelihood of being a primary sexual abstainer compared to being currently sexually active by 86%. Likewise, discussing sexual issues with fathers decreased the likelihood of being a secondary sexual abstainer relative to being currently sexually active by 92%. Thus, compared to those that did not discuss sex-related issues with only fathers, youth who discussed sexual concerns with only their fathers were significantly less likely to be primary sexual abstainers or secondary abstainers than the currently sexually active.

Finally, sexual communication with only friends also decreased the relative likelihood of being a primary sexual abstainer than currently sexually active by 89%. The effect of sexual discussion with only friends persisted when the secondary abstainers were compared to the currently sexually active. Discussing sexual issues with only friends decreased the likelihood of being a secondary sexual abstainer relative to the currently sexually active by 83%. Therefore, youth who discussed issues about sex with only their friends, compared to those who did not, were less likely to be primary sexually abstinent or secondary abstinent compared to being currently sexually active.

## Discussion

The study examined the predictors of primary and secondary sexual abstinence among never-married youth in urban poor Accra. The results showed that primary sexual abstinence was relatively rare among the youth, with only about one-fifth indicting that they had never engaged in sexual intercourse. Primary abstinence was especially less likely among females. This finding is consistent with the pattern observed nationally [4]. Results from the 2014 Ghana Demographic and Health Survey showed that 22% of unmarried females aged 20–24 years were primary abstainers compared to 26% of males. This suggests that many unmarried female youth in the country are engaging in sex with older male sexual partners [23]. The fact that the percentage of female primary abstainers (17.7%) in this study is significantly lower than the percentage nationally (22%) suggest that female youth in urban poor Accra are more likely to engage in sexual intercourse with older male partners than nationally. Sexual relationships with older male partners limit young women’s capacity to negotiate safe sex and increase the risk of unwanted pregnancies and STIs including HIV [36].

Regarding secondary abstinence, the study found that it was widely practiced with almost half of the sample indicating that their last sexual intercourse was more than a year ago. This suggests that many youth can abstain from sex for a relatively long period after sexual debut. Such youth should therefore be included in programmes aimed at promoting abstinence behaviour. It is also entirely possible that many of the secondary abstainers engaged in sporadic sex, some of which is likely to be unprotected. Thus, secondary abstainers who engage in sporadic sex should be encouraged to use appropriate modern contraceptives for protection during such irregular sexual encounters.

Overall, this study identified six factors that predicted primary and secondary abstinence: gender, schooling, religion, informal social support to households, locality of residence and sexual communication. It is important to note that only sexual communication predicted both primary and secondary abstinence, emphasizing the importance of communicating with youth about matters that might even be considered delicate. The other five factors only predicted primary abstinence, but not secondary abstinence. The fact that only sexual communication factors predicted secondary abstinence implies that open communication about sex especially with parents is an effective way of inducing abstinence after sexual debut among unmarried youth.

One of the main findings of this paper is that females are less likely to be primary sexually abstinent compared to males. Even though studies in other countries show that females are more likely to be primary sexual abstainers, the pattern in Ghana is the reverse. The finding in this study is therefore in line with previous studies in the country [22]. In Ghana and across much of Africa, young unmarried females usually face pressures to engage in sex with older male partners for financial and social support, and the cultural norms subtly encourage such sexual relationships. This is especially the case in areas with limited access to basic resources such as urban poor settings. This increases the susceptibility of unmarried urban poor female youth to unwanted pregnancies and STIs including HIV. Therefore, efforts to improve sexual and reproductive health outcomes among urban poor youth in Accra need to prioritise females and also focus on addressing factors such as poverty and cultural norms which encourage females to engage in sex for survival.

With regard to schooling, the youth in school were more likely to be primary sexual abstainers than currently sexually active compared to their out of school peers. This finding is consistent with studies across different settings, which show that schooling promotes sexual abstinence and delays sexual debut [13, 37, 38]. Kirby argues that schooling reduces the amount of idle time youth have, increases their education and career aspirations, and empowers them with the necessary skills to reject unwanted sex [37]. This implies that increasing school enrollment and ensuring that urban poor youth do not drop out of school could be an effective strategy of promoting sexual abstinence. Schools could also provide youth in urban poor settings with accurate information about sexual and reproductive health issues such as fertility awareness, sexual intercourse, pregnancy and STIs.

In addition to schooling, attaching importance to religion positively predicted primary sexual abstinence. The youth who reported that religion was very important to them were more likely to be primary sexually abstinent than currently sexually active. This finding is consistent with the results of previous studies [39–41]. A recent study among Nigerian youth found that those who were highly religious were 81% more likely to abstain from sex compared to their peers with low levels of religiousity [41]. Youth who attach importance to religion tend to have negative attitudes to premarital sex, and perceive a higher pressure to avoid sex than those that do not. Given the effect of religion, it is possible to reinforce sexual abstinence and safe sex among urban poor youth through religious institutions. Thus, efforts should be made to build the capacity of religious leaders in such settings to provide comprehensive sexuality education to adolescents and youth.

Studies examining the link between social support and sexual abstinence are quite rare. Social support involves the transfer of material and social resources. Given the overwhelming evidence that urban poverty reduces the ability of youth to negotiate safe sex including abstinence, it is expected that access to social support will increase the likelihood of sexual abstinence. The results of this study corroborate the expected positive relationship between access to social support and sexual abstinence. Youth residing in households that received informal social support were more likely to be primary sexual abstainers than currently sexually active. Social support in the form of material resources or cash to households probably reduces the pressure on youth in those households to engage in sex for survival. One experimental study found that female adolescents who received cash transfer were less likely to engage in unprotected sex than those that did not [33]. Intangible social support in the form of emotional support may also increase the self-confidence, self-esteem and self-efficacy of youth to abstain from sex. By implication, social support programmes such as cash transfers to vulnerable households in urban areas could delay sexual debut and reduce the risk of unwanted pregnancies and STIs.

Furthermore, the study corroborates the negative association between slum residence and sexual abstinence [9, 11]. Youth in Agbogbloshie, the slum community, were less likely to be primary sexually abstinent than currently sexually active compared to those in Ussher Town. Even though all the three study communities are classified as urban poor, Agbogbloshie is a slum with many dwelling units being wooden shacks. Agbogbloshie also has limited spaces for recreational activities. Moreover, because Agbogbloshie is predominantly a migrant community, it may have weak traditional institutions and mechanisms for regulating youth sexual behaviour. These social conditions may explain why youth in Agbogbloshie are less likely to be primary sexual abstainers compared to their peers in Ussher Town. It is therefore pertinent to prioritise slums such as Agbogbloshie in interventions aimed at delaying sexual debut. Such interventions should focus on improving the social conditions of slums such as providing housing, health services and access to recreational facilities.

Regarding sexual communication, the results indicate that talking to both parents about sex increases the likelihood of being a primary sexual abstainer or secondary abstainer relative to being currently sexually active. There is evidence that when parents openly discuss issues of sex with their children, they are more likely to abstain from sex or practice safe sex [25, 42, 43]. Sexual communication with both parents is indicative of maximum support for children. Discussing issues of sex with both parents could also provide youth with adequate information about sex, pregnancy, sexually transmitted infections and socialize them into attitudes and norms that value abstinence. It is important for parents to understand that providing accurate information about sex to their children will help them to make informed and healthy choices. Thus, efforts to delay sexual debut and improve sexual and reproductive health outcomes among urban poor youth in Accra need to encourage open discussion about issues of sex between youth and their parents. Parents also need accurate information about fertility, reproduction, contraception, STIs and HIV, to be able to have meaningful discussions with their children.

While sexual communication with fathers has been shown to delay sexual debut in other studies [44], a contrary pattern emerged in this study. The study found sexual communication with only fathers to predict a decreased likelihood of both primary and secondary abstinence. Fathers probably engage in discussions about sex with their youth when they suspect the youth have become sexually active [27]. The sexual activity may therefore have preceded the communication with fathers. Moreover, sexual communication with only fathers may also be symptomatic of family dysfunction, which increases the likelihood of sexual intercourse among youth. Future studies need to assess the timing, medium and style of sexual communication between youth and their fathers. This may help to explain the counter-intuitive finding in this study that sexual communication with fathers decreases the likelihood of primary and secondary abstinence.

Finally, the study also found that sexual communication with only friends is associated with a lower likelihood of primary and secondary abstinence relative to current sexual activity. This finding is consistent with the results of studies conducted elsewhere [45, 46]. Friends and peers of youth usually have permissive attitudes to premarital sexual intercourse. Thus, sexual communication with only friends probably provides youth with information that encourages sexual experimentation. It is entirely plausible that youth are choosing to discuss their sexual experiences with only friends after having initiated sexual intercourse. While sexual communication with friends and peers needs to be encouraged and supported, it is important to ensure that information shared through such channels are accurate, and discourage risky sexual experimentation.

### Limitations of the study

Even though this study relied on high quality secondary data, it has a number of limitations. One of the main limitations is the narrow conceptualisation of sexual intercourse as penile-vaginal intercourse. Such a narrow conceptualisation excludes oral and anal intercourse, which could also expose youth to STIs and HIV. We could not include oral and anal intercourse in our measurements because the surveys we relied on did not collect such data. Also, the surveys did not collect data on the psychological predictors of sexual abstinence as outlined in the integrative theoretical framework. Another limitation of the study is the use of cross-sectional data. Even though we pooled data from two surveys at different times, the data is essentially cross-sectional and does not allow for causal inferences to be made from the results, because of the difficulty of time-sequencing events. Despite these limitations, this study is one of the few to simultaneously examine the predictors of primary and secondary abstinence among youth in an urban poor context in sub-Saharan Africa. In addition, the findings of this study provide policy makers and reproductive health practitioners with factors that need to be considered when developing policies and interventions to delay sexual debut and reduce unwanted pregnancies, STIs, and HIV among youth in urban poor settings in the sub-region. Furthermore, the study used robust data and analytic techniques to examine sexual abstinence in a context where data is often unavailable or of questionable quality.

## Conclusion

In summary, this study found that primary sexual abstinence was quite uncommon among unmarried youth age 20–24 years in urban poor Accra. This was especially the case among females. Secondary abstinence was, however, widely practiced among the youth in this study. The study revealed that primary sexual abstinence was predicted by gender, schooling, religion, household access to social support, source of sexual communication and locality of residence. Thus, being female, out of school, discussing issues about sex with only fathers or friends and residing in the slum (Agbogbloshie) significantly predicted a lower likelihood of primary sexual abstinence. However, residing in a household that received social support and discussing issues about sex with both parents significantly predicted a higher likelihood of primary abstinence. Regarding secondary abstinence, it was only the source of sexual communication that significantly predicted it. Discussing issues about sex with both parents predicted a higher likelihood of secondary abstinence while discussing issues about sex with only fathers or friends predicted a lower likelihood of sexual abstinence. The findings of this study suggest that efforts to promote primary and secondary abstinence in urban poor Accra need to consider these factors which range from individual through household factors to the locality of residence. Focusing efforts on one of these factors without the others may fail to yield the desired benefits of delaying sexual debut and encouraging secondary abstinence. Despite the importance of all the predictor factors, the study revealed that sexual communication with both parents was the only factor that predicted a higher likelihood of both primary and secondary sexual abstinence. We therefore recommend open and honest discussion about issues of sex between youth and their parents as a key strategy for promoting sexual abstinence and safe sex among urban poor youth in Ghana.

## Data Availability

Data from the Urban Health and Poverty Surveys are available at the Regional Institute for Population Studies, University of Ghana upon a written request and with approval from the Institute.

## Abbreviations

ABC: Abstain, Be faithful and use Condoms (ABC)
EAs: Enumeration Areas
NMIMR-IRB: Noguchi Memorial Institute for Medical Research-Institutional Review Board
RIPS: Regional Institute for Population Studies
STIs: Sexually Transmitted Infections
